# A phase III trial to evaluate the efficacy, fabric integrity and community acceptance of Netprotect® using a recommended long-lasting insecticidal net as positive control

**DOI:** 10.1186/1475-2875-13-256

**Published:** 2014-07-07

**Authors:** Karel Van Roey, Siv Sovannaroth, Tho Sochantha, Mao Srey Touch, Olivier Pigeon, Vincent Sluydts, Lies Durnez, Marc Coosemans

**Affiliations:** 1 Department of Biomedical Sciences, Institute of Tropical Medicine of Antwerp, Nationalestraat 155, B-2000 Antwerpen, Belgium; 2 National Center for Malaria Control, Parasitology and Entomology, Phnom Penh, Cambodia; 3 Agriculture and Natural Environment Department, Walloon Agricultural Research Centre (CRA-W), Gembloux, Belgium; 4 Department of Biomedical Sciences, Faculty of Pharmaceutical, Veterinary and Biomedical Sciences, University of Antwerp, Universiteitsplein 1, B-2610 Wilrijk, Antwerp, Belgium

## Abstract

**Background:**

The evaluation of new long-lasting insecticidal bed nets (LLINs) is coordinated by the WHO Pesticide Evaluation Scheme (WHOPES). In 2007, Netprotect® was granted WHOPES interim recommendation after Phase I and II evaluations. Present study evaluates Netprotect® in a Phase III trial in rural Cambodia.

**Methods:**

A randomized, prospective longitudinal study design was used to assess the performance of Netprotect® over a period of three years, using conventionally-treated nets and a WHOPES recommended LLIN (PermaNet® 2.0) as positive controls. The primary outcomes were the physical integrity, insecticide content and cone bioassay performance using.

**Results:**

The baseline deltamethrin concentration of 43% of Netprotect® nets were below the tolerance limit while 27% of PermaNet® 2.0 nets were above the target dose limits. By 36 months Netprotect® retained 35% while PermaNet® 2.0 retained 49% of baseline insecticide dose. Moreover the proportion of the inactive deltamethrin *R*-alpha isomer in the Netprotect® nets was 33% at the baseline and increased to 69% after three years while it was low and almost constant for PermaNet® 2.0 (3-7%). Only 71% of Netprotect® met the WHO criteria for bio-efficacy after three years while at least 80% is required. Moreover Netprotect® nets failed for the WHOPES criteria after 12 and 24 months. The reference LLIN met the WHOPES criteria throughout the study. Over the entire three years the reference LLIN did obtain significant higher mosquito mortality than Netprotect®. The physical integrity was based on the proportionate hole index and after three years, 25% of Netprotect® and 30% of PermaNet® 2.0 were in a mediocre or poor state.

**Conclusion:**

Netprotect® did not meet the minimum WHO criteria for bio-efficacy after 12, 24 and 36 months. The use of a reference LLIN as positive control was helpful for data interpretation. However, for future three-year studies, it is essential that before initiating any study nets should be checked for their specifications and this for both the candidate LLIN as well as for the reference LLIN. Moreover, to improve the accuracy of the success rate of the candidate LLIN more nets should be tested for their bio-efficacy at the end of the trial.

## Background

Over the past decades, insecticide treated nets (ITNs) have proven to be the key measure in malaria prevention worldwide [1-3]. This success is mainly related to the development of long-lasting insecticidal nets (LLINs) where insecticide is incorporated into or coated onto the fibres. An LLIN must retain its biological activity after at least 20 washes and three years of use under field conditions. So far the World Health Organization (WHO) did not define criteria for net integrity but has invited the national malaria control programmes to assess the durability of LLINs in operational conditions [4].

New LLINs are granted a time-limited WHOPES interim recommendation for use after successful evaluations in lab conditions (Phase I) and in experimental huts (Phase II), during which wash-resistance, induced vector mortality and blood-feeding inhibition is demonstrated. To receive a full recommendation, prospective studies (Phase III) are required to demonstrate a bio-efficacy during at least three years. Physical durability and survivorship of LLINs are also assessed at this stage.

Currently seven LLINs have received full recommendation from WHOPES and four have an interim recommendation. Until recently candidate LLINs were compared to conventionally-treated nets (CTNs). However the inter- and intra-net variation of the amount of insecticide was considerable with these CTNs and in 2012 the WHOPES working group decided to use a reference LLIN as positive control [5].

The present study evaluates Netprotect®, interim recommended since 2007 [6], in a phase III trial in Cambodia according to WHO guidelines and procedures applicable at the onset of the study using a CTN as positive control [7]. Moreover, a reference LLIN (PermaNet® 2.0) was used as additional positive control. The performance of the nets was assessed in terms of efficacy, insecticide content, wash resistance, longevity, fabric integrity and community acceptance under field conditions over a period of three years.

## Methods

### Study area

Present study was conducted between 2009 and 2013 in Veal Veng district, a rural forested area in the province of Pursat, in Cambodia at the border with Thailand. In this area, 1.5% malaria prevalence is detected based on Rapid Diagnostic Tests/microscopy (Cambodia Malaria Survey 2010) [8]. The main malaria vectors are *Anopheles dirus sensu stricto*, *Anopheles minimus s.s., Anopheles maculatus* and *Anopheles barbirostris*. All vectors are fully susceptible to pyrethroids [9].

In Cambodia, the overall proportion of people sleeping under an ITN/ LLIN “the previous night” increased the last years to more than 50%. About 63% of households owned sufficient amount of mosquito nets but only 23% of the 3,164 households (HHs) interviewed during the Cambodia Malaria Survey (CMS) 2010 [8] owned sufficient LLINs. This net coverage contributed to the decrease of the overall malaria prevalence in Cambodia from 4.4% in 2004 [10] and 2.6% in 2007 [11] to 0.9% in 2010.

### Study design and sample size

A phase III field trial, designed as a prospective longitudinal study, was set up to study the performance of Netprotect® using conventionally-treated nets (CTN) as a positive control. The study protocol followed the WHO guidelines for phase III LLIN field trials [7] except that a reference LLIN (PermaNet® 2.0) was used as an additional positive control. The unit of observation was the household (HH), in which one net was assigned for follow up. CTNs and their use were monitored for one year and the LLINs (Netprotect®, PermaNet® 2.0) for three years.

Seven field surveys were organized for the follow-up of the nets. A first survey took place one week post-net distribution and subsequent surveys were conducted every six months. During each survey a randomized sample of the three net types was collected and submitted to a physical integrity test and cone bioassay test. In the latter test, susceptible colony mosquitoes were exposed to the nets and the mosquito knockdown and mortality was recorded (see Bio-efficacy section for details*)*. A minimum of 510 HHs was necessary enabling sampling 30 nets of all types per survey. To count for nets lost for follow up, 90 HHs for each LLIN type and 50 HHs for CTN were added. Therefore, seven villages (with a minimum of 90 HHs per village) were selected in Veal Veng district to cover a total of 740 HHs.

### Nets, CTN treatment and distribution

The LLIN Netprotect®, manufactured by Bestnet Europe LTD, is a LLIN made of polyethylene with deltamethrin incorporated into the 118-denier monofilament with a target dose of 1.8 g active ingredient (a.i.)/kg, corresponding to 68 mg a.i./m^2^ (weight 35 g/m^2^). The nets distributed had a standard size of 160 cm W × 180 cm L × 150 cm H. The PermaNet® 2.0 was bought from Vestergaard Frandsen. This is a WHOPES fully recommended LLIN [12] made of knitted poly-filament polyester fibres coated with deltamethrin with a target dose of 1.4 g a.i./kg for a 100 denier yarn, or 55 mg a.i./m^2^. The PermaNet® 2.0 nets had the same colour (blue) and size as the Netprotect® nets. The conventionally-treated nets (CTNs) were blue but slightly wider (190 cm W × 180 cm L × 150 cm H) than both LLINs with a fabric weight of 31.1 g/m^2^. The deltamethrin treatment (Deltamethrin EC 10 or 10,0 g a.i./L) of these nets, as per the standard WHO instructions [13], was carried out by two trained dippers provided with adequate protective gear, at a target dose of 25 mg a.i./m^2^.

Prior to the net distribution, a HH census was conducted in the study area to obtain the demographic and socio-economic characteristics of each HH. The HH census data were entered in an Access database to form a master list. From this master list, all HHs in the selected villages were randomly allocated to one of the three study arms in a 2:2:1 ratio, for respectively Netprotect®, PermaNet® 2.0 and CTN, and this in each village. For HHs assigned to Netprotect® or PermaNet® 2.0 nets, enough nets of the same type were distributed to assure full coverage (i.e. one net per two inhabitants), but only one of these nets was assigned as net to follow up. In HHs assigned to CTN, only one CTN was distributed, and additional Netprotect® nets were distributed to cover all residents. In December 2009, a total of 148 HHs were provided with a CTN, 305 HHs with Netprotect® nets and 309 HHs with PermaNet® 2.0 nets.

All distributed nets received a unique Net ID number, written with wash-resistant ink on the factory label. The net with the highest code number within each HH was retained for follow up.

### Surveys/field procedures

For every sampling round a HH randomization was made to sample 30 nets of each study arm, evenly distributed over the villages. As people were not always present during the survey or nets were lost, 60 HHs per study arm were randomized. The HHs were visited according to the randomization order, until the number of samples were achieved per village and type of net brand. Nets selected and withdrawn were replaced with a new LLIN without ID number. HHs where people were absent (for more than three consecutive days) were maintained in the master list for the next randomization, while the HHs where the net was sampled were removed from the master list. In the first year of the study the HHs that recorded loss of their net for follow up were also removed from the master list. However, because of the high number of nets lost for follow up, it was decided after one year that only the HHs that were sampled or moved away would be removed from the master list. Nets randomized for the second time and still lost for follow-up, were recorded as lost and removed from the master list. From that point on though, the second highest net ID in that specific HH would become the new net to follow up for the upcoming sampling surveys.

During the surveys, HH owners or net users were interviewed by trained staff about their net use (method and frequency), washing habits (frequency and method of washing and drying) and the occurrence of adverse events. The net to follow up was sampled for submission to physical integrity tests and cone bioassay tests. Chemical analysis were done only on the nets sampled 1 week, 6 months, 12 months and 36 months after net distribution. At the end of the first study year, besides the normal sampling of the three net types, the remaining CTNs were collected and replaced with a Netprotect® or PermaNet® 2.0.

In the annual surveys (at the end of months 12, 24 and 36) in addition to the randomized HHs, all households remaining in the master list were visited door-to-door. Whereby the physical presence/absence and the information on people’s net perceptions and practices were recorded, to help estimate the annual attrition rate.

### Physical inspection of nets

All nets sampled during the surveys were examined for their physical integrity. The integrity of the nets was determined counting the number of tears and holes as described in [14]. Hole sizes were categorized in three groups; holes allowing a thumb to pass through, holes between a thumb and a closed fist and holes bigger than a closed fist. A proportionate hole index (pHI) [15,16] was calculated by making the sum of the holes weighted by size for each net. For these groups, the weights used to calculate the pHI were 1, 9 and 56 as described in [15,16]. To better translate the hole index to an integrity status (net condition) for each sampled net, the pHI is categorized into good (%25), fair (25–174), mediocre (175–299) and poor (>299) net condition [16]. The dirtiness of the nets was determined by comparing the nets to reference samples (clean, bit dirty, dirty, very dirty and never used).

### Insecticidal activity

#### Bio-efficacy

Five samples per net (25 × 25 cm) were cut from the positions 1 to 5 of the net according to the guidelines [4] once the physical integrity test was completed. The WHO cone bioassays, as described in the WHOPES guidelines [7], were conducted on these samples.

Bioassays were conducted at 27 ± 2°C and 80 ± 10% relative humidity. The *An. dirus s.s.* colony used in these bioassays was fully susceptible to deltamethrin, DDT and permethrin. Five non-blood-fed, 2–5-day-old *An. dirus* females were exposed for three minutes in each cone and then held for 24 hours with access to a sugar solution. For each sample two replicates were performed. Knock-down and mortality were measured 60 minutes and 24 hours after exposure respectively. A negative control, from an untreated net, was included in each round of cone bioassay testing. If the mortality in the control, insecticide free net, was below 10% for a given day the data were adjusted with Abbott’s formula [17]. If the mortality in the control was >10%, all the tests for that day were repeated.

The nets sampled one week after distribution (Survey 1) were used to assess the base line profile for bio-efficacy of insecticide. Based on the cone bioassay results at month 36, the LLINs that did not meet the criteria of ≥95% knockdown or ≥80% mortality were subjected to a tunnel test to determine the efficacy (mortality and blood feeding inhibition) as described in the WHOPES guidelines [7]. As the colony of *An. dirus* is maintained on mice, this host was preferred to guinea pigs for the tunnel test. Nets fulfilling the criteria of ≥80% mortality or ≥90% blood-feeding inhibition in the tunnel test were considered to be still effective. The tunnel test was valid only if in the control a threshold of 36% blood-feeding is reached [14]; however, higher rates such as 50% are more appropriate [4].

#### Insecticide content

Nets sampled at 1 week, and 6, 12 and 36 months post-distribution were chemically analysed to determine their insecticidal content. This was carried out on four pieces (25 × 25 cm) per net, similar to the bioassay samples on positions 2 to 5 of the net. Each sample was individually packed in aluminum foil and kept at 4°C until further analysis at the Walloon Agricultural Research Centre (CRA-W), Gembloux, Belgium (WHO Collaborating Centre for Quality Control of Pesticides) using an ISO 17025 accredited analytical method.

The four samples from each LLIN/CTN were combined to provide the average concentration of the insecticide in each LLIN/CTN. Briefly, deltamethrin was extracted by heating the sample under reflux for 60 minutes with xylene in presence of dipropyl phthalate as internal standard. Insecticide content determination was carried out by using Gas Chromatography with Flame Ionization Detection (GC-FID).

Results were expressed as g a.i./kg and converted to mg a.i./m^2^ using the fabric weight. A tolerance limit of ± 25% of the target dose is recommended in the WHO specification [18]. This analytical method was fully validated for determination of deltamethrin in incorporated LLINs and was proved to provide similar results than those obtained by the standard reference CIPAC method 333/LLIN/(M2)/3 (HPLC-DAD) published later in 2012 in the CIPAC Handbook N [19].

The content of both the biological active S-alpha isomer (deltamethrin) [(S)-a-cyano-3-phenoxybenzyl (1R,3R)-3-(2,2-dibromovinyl)-2,2-dimethylcyclopropane carboxylate)] and the inactive R-alpha isomer [(R)-a-cyano-3-phenoxybenzyl (1R,3R)-3-(2,2-dibromovinyl)-2,2-dimethylcyclopropane carboxylate)] was measured on all samples.

### Functional survivorship rate

The CTN and LLIN functional survivorship rates were calculated from the records made during the surveys at the end of each study year. The functional net survivorship accounts for loss due to damage only and is not influenced by nets that are removed from the study area (stolen, given away or family moved) as they still may be functional [20].

### Data entry and analysis

All data were recorded on standard forms. The hard copy data was single entered into the main Access database. Data entry was checked by cross checking all entered records of adverse events, physical integrity and bioassays with the hard copy files. All data were imported into R, version R-3.0.2 [21], for statistical analysis.

Descriptive analyses were done on the demographic household census data, to define the household characteristics. Data collected on net use and net washing habits were analysed using a chi-square test (χ^2^) for significance of deviation between the net types.

For the analysis of net performance (physical condition, chemical content, bioassay) all sampled nets were used except nets that were still in the original plastic bag (i.e. never used) and nets that were completely torn and thrown away.

For each net chemical content and bioassay performance were calculated from all samples apart from position 1, as the lowest part of the net may be exposed to excessive abrasion in routine use. For chemical analysis an inter-net variation was expressed by relative standard deviation (RSD). For the bioassays, each of the four samples were tested individually and an assessment of between-net variability was done with a RSD. The within-net variability was expressed as the difference of samples of the same net to the mean expressed as percent of the mean and then averaged over the sample [15].

A Chi-Square test over multiple strata was conducted (Mantel Extension Test [22] on the acceptance of the nets (proportions of LLINs that fulfil the WHO criteria (≥95% knockdown or ≥80% mortality).

A generalized linear mixed model (GLMM) was fitted for chemical content, bio-efficacy and net integrity as response variable. As fixed effect explanatory variables the net type, survey (time point), net condition, dirtiness of the net, net use intensity, number of washes, drying and washing method and socioeconomic status (SES) were included. The net ID was included as a random effect. Per outcome the model was optimized by removing the insignificant variables using a likelihood ratio test [23] or by comparing the AIC (Akaike information criterion) values [24].

### Ethical clearance

Ethical approval for the study was obtained from the Ethics Review Committee of WHO for two arms (CTN and Netprotect®) and from the Ethical Committee of Cambodia, the Institutional Review Board of the Institute of Tropical Medicine and the legal ethical committee of the University of Antwerp for all three arms (CTN, Netprotect® and PermaNet® 2.0).

An informed consent was signed by impregnators and by HH owners. All participants were informed about the benefit of using an insecticide impregnated net and the additional advantages of using a LLIN. The information sheet addressed also the possible adverse effects and their remediation for all users.

## Results

### Net distribution and household characteristics

The random allocation of each HHs to one of the net types was in all 7 villages according to a 2:2:1 ratio, for respectively Netprotect® (305 HH), PermaNet® 2.0 (309 HH) and CTN (148 HH). All HHs together comprised of 3,832 individuals of which 45% were younger than 17 years and 26% were between 17 and 30 years old. The average age of the head of the HHs is 42 years, 74% of them had no education, 23% a primary education, and 87% of head of HHs are farmers. 82% of the houses were built on stilts compared to 18% built on the ground, the walls are made of wood (78%) or thatch (21%) and the roofs are covered with sheets of corrugated iron (61%) or thatch (32%) (Additional file 1).

### Net surveys, net use and washing habits

During 8 field surveys spread over the three study years, 1435 HHs were visited. In the first two years of the study, more than 80% of the HHs declared to use the net year-round and every night (Additional file 2). The remaining 20% of the HHs only used their net seasonally, did not use them or did not provide an answer. No significant differences in net use intensity were found after one year between the three study arms (χ^2^ test: P = 0.173, df = 12). After three years the net use intensity dropped to about 60% for both Netprotect® and PermaNet® 2.0. Moreover, 34% of Netprotect® and 20% of PermaNet® 2.0 were recorded as not used anymore and stored away. Those net users replaced these nets with up-to-that-point unused spare nets. During the first year 30/601 nets of all three net types were kept as spare nets. This occurred because of the over saturation of nets or because most families slept with more than two individuals under one net. Similarly no significant differences in net use intensity were found between the two LLINs after three years (χ^2^ test: P = 0.213, df = 3).

Out of 522 HHs in which the net to follow up was present at the end of each year, 250 (48%) HHs recorded to have washed the net (Additional file 3). For net that have been washed, the average number of washes per year reported by the HH for Netprotect and Permanet 2.0 was 3.4 and 3.5 respectively for year one; 1.7 and 2.1 for year two, and 1.3 and 2.4 for year three. The accuracy on the reported washing behaviour is however questionable as the reported washes declined for the last survey. Irrespective of the net type, almost all nets were washed with cold water (98.8%) and with a local detergent or soap (96%). In 8% of cases the nets were rubbed against rocks. A total of 65% of the washed nets were dried outside in the sun, 23% were dried outside in the shade and 10% were dried inside.

### Declared adverse events among the CTN impregnators and CTN/LLIN users

Both impregnators declared to experience a bad smell during impregnation, no other adverse events were recorded during or after impregnating the nets. Net users from all study arms declared some adverse events during the first 6 months of use, while from 12 months post net distribution and onwards no more adverse events were declared. The most frequently declared adverse events were bad smell, itching skin and eye irritation. Analysing the declared adverse events among the three study arms within each survey, no significant differences were found between the three net type users.

### Net functional survivorship

The functional survivorship stayed above 90% for both LLINs in the first two years but after three years this dropped to about 60% for both Netprotect® (58.1%) and PermaNet® 2.0 (61.2%) (Table 1).

**Table 1:** Functional net survivorship rate

**Survey**	**Net type**	**N**	**Functional survivorship (95% CI)**
**Year 1**	CTN	45	97. 8 (88.4 – 99.6)
	Netprotect®	141	96.5 (91.5 – 98.2)
	PermaNet® 2.0	143	98.6 (95–99.8)
**Year 2**	Netprotect®	67	92.5 (83.7 – 96.8)
	PermaNet® 2.0	78	98.7 (93.1 – 99.8)
**Year 3**	Netprotect®	43	58.1 (43.3 – 71.6)
	PermaNet® 2.0	49	61.2 (47.2 – 73.6)

Calculated from all the HHs visited at the end of each study year where the net was present or discarded because of being torn or burned (Surveys 4, 6 and 8). HHs where the inhabitants were absent or where the net was recorded as given away, used elsewhere, stolen or lost were not included in the estimates of survivorship.

### Physical condition

During the three years of follow up, a total of 507 nets were sampled and all tested for their physical integrity. However 42 nets were excluded from analysis, of which 33 had never been used and nine had been thrown away because being completely torn (one CTN, three Netprotect® and five PermaNet® 2.0).

**Figure 1:**
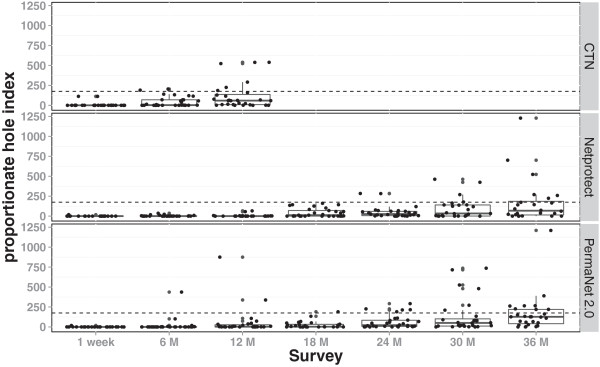
**Physical condition of individual nets based on the proportionate hole index (pHI).** Box plot indicating median and interquartile range (0.25–0.75), dashed line = threshold mediocre/poor net condition (pHI ≥175).

The proportion of nets with at least one hole increased rapidly within the first year up to 89%, 50% and 63% of respectively CTN, Netprotect® and PermaNet® 2.0. The increase continued up to two years of use for both LLINs to 86%. Finally after three years 83% of Netprotect® and 93% of PermaNet® 2.0 had at least one hole. Similarly the proportionate hole index (pHI) increased rapidly over time, but important variation exists between nets for respectively all net types. Consequently the median and interquartile range ([IQR] 0.25–0.75) were used to present the pHI (Figure 1 and Additional file 4). After one year of use the median pHI was 59 (IQR 8–137) for CTN, 0.5 (IQR 0–3) for Netprotect® and 3 (IQR 0–27) for PermaNet® 2.0. After three years, the pHI increased to 68 (IQR 14–186) and 127 (IQR 41–220) for respectively Netprotect® and PermaNet® 2.0, of which 25% of Netprotect® nets. By the end of the study, the percentage of nets categorized as in mediocre or poor condition (pHI ≥ 175) were 25% and 30% for Netprotect® and PermaNet® 2.0, respectively. Over the entire three-year follow-up no significant difference was found between both LLIN’s physical durability (Odds ratio [OR] = 0.82, 95% CI 0.49–1.38 - GLMM, P = 0.45). Net integrity of both Netprotect® and PermaNet® 2.0 were affected by the dirtiness, with significantly more holes recorded in dirty and very dirty nets (OR = 39, 95% CI 17–92 - GLMM, P % 0.001 and OR = 122, 95% CI 47–315 - GLMM, P % 0.001, for Netprotect® and PermaNet® 2.0 respectively).

### Chemical content and insecticidal retention

**Figure 2:**
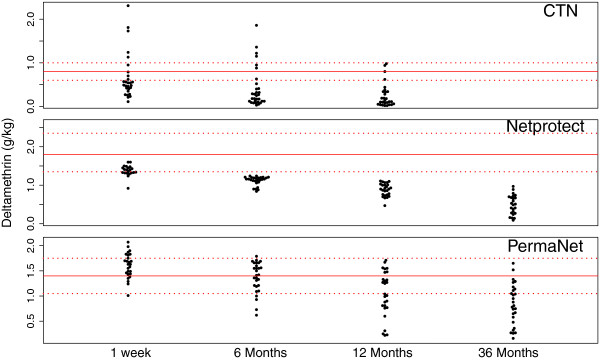
**Deltamethrin content ( *S *-isomer) on individual sampled nets over time.** Full line = the target dose, dashed lines = the tolerance limits (±25% of target dose), CTN = conventionally-treated net.

The average active deltamethrin content of each of the three net types measured one week post distribution was within the given target dose, though for CTN and Netprotect® only marginally (Table 2A). Only 14% of the sampled CTNs were actually within its chemical target dose and this with a high between-net variation (RSD: 78%) showing a low homogeneity of treatment (Figure 2 and Table 2A). Regarding the LLINs, 57% of Netprotect® nets (RSD: 10%) and 69% of PermaNet® 2.0 nets (RSD: 15%) were within the target dose. The remaining 43% of Netprotect® nets were below the threshold (Figure 2). For PermaNet® 2.0 nets, 4% were below the tolerance limit and 27% were above the maximum allowed deltamethrin content. After six months of use, all sampled Netprotect® nets dropped below the initial chemical target dose while 48% and 33% of PermaNet® 2.0 nets sampled, respectively after one and three years, were still within the limits of the initial target dose. Despite this difference, and taking into account the insecticide content after one week as baseline, the deltamethrin retention at 6, and 12 months was similar for the two LLINs (81% and 62% retention for Netprotect®; 86%, 65% for PermaNet® 2.0). Though a difference was noted after three years, 36% retention for Netprotect® and 49% for PermaNet® 2.0. The CTNs sampled after 12 months of use retained only 37% of its original content.

**Table 2:** Chemical analysis

**A**		**Average insecticidal residue g/kg (RSD%)**						
		**% nets within the tolerance limits of the target dose**						
	**N**	**1 week**	**N**	**6 months**	**N**	**1 year**	**N**	**3 years**
**CTN**	28	0.68 (77.8)	31	0.41 (110.2)	28	0.25 (109.6)		
		14.3		9.7		14.3		
**Netprotect**®	21	1.38 (10.3)	27	1.12 (10.6)	26	0.88 (18.6)	24	0.49 (51.4)
		57.1		0		0		0
**PermaNet**® **2.0**	26	1.61 (15.3)	28	1.37 (22.2)	27	1.05 (43.4)	27	0.83 (49.2)
		69.2		82.1		48.1		33.3
**B**	**Deltamethrin *R*-alpha isomer (insecticidally inactive) content, expressed as percentage of the deltamethrin content (range g/kg)**							
**Survey**	**1 week**		**6 month**		**1 year**		**3 years**	
**CTN**	3%		1%		20%			
	(%0.01 to 0.05 g/kg)		(%0.01 to 0.01 g/kg)		(0.01 to 0.39 g/kg)			
**Netprotect**®	33%		51%		53%		69%	
	(0.35 - 0.54 g/kg)		(0.46 - 0.66 g/kg)		(0.20 - 0.57 g/kg)		(0.09 - 0.55 g/kg)	
**PermaNet**® **2.0**	7%		3%		6%		3%	
	(0.06 to 0.14 g/kg)		(0.01 to 0.06 g/kg)		(0.03 to 0.09 g/kg)		(%0.01 to 0.05 g/kg)	

A. Average deltamethrin (*S*-Isomer) content (in g/kg). The target doses at baseline (tolerance limits of ±25%) are 0.8 (0.6-1) g/kg, 1.8 (1.35 -2.25) g/kg and 1.4 (1.05–1.75) g/kg for CTN, Netprotect® and PermaNet® 2.0 respectively. Between-net variation is expressed by the Relative Standard Deviation (RSD%).

B. Deltamethrin *R*-alpha isomer content.

Additionally to the content of active *S*-alpha isomer (deltamethrin), the content of the deltamethrin *R*-alpha inactive isomer was measured per survey and net type. In the baseline CTN samples 3% of the deltamethrin content counted for the *R*-alpha isomer. This amount remained below 3% in the samples after six months but did increase to 20% in the samples collected after one year (Table 2B). For Netprotect® the deltamethrin *R*-alpha isomer content in the baseline samples was 33% of the deltamethrin content; this high percentage even increased in the samples collected after 6, 12 and 36 months up to respectively 51%, 53% and 69% of the deltamethrin content. For PermaNet® 2.0 the deltamethrin *R*-alpha isomer content in the baseline samples was 7%; this amount remained low in the samples collected after 6, 12 and 36 months, respectively 3%, 6% and 3% of the deltamethrin content.

### Bio-efficacy

Results from WHO cone bioassays are presented in Table 3. Mortality in the negative control never exceeded 4%, this occurred only in two bioassay tests and in another eight tests control mortality reached 2% while no mosquito mortality was recorded in all other 451 bioassay tests. The CTNs were fully effective after six months of use, but both the mosquito knockdown and mortality rates of CTNs dropped sharply after one year of use to 56% and 64% respectively. For both LLINs the average knockdown and mortality were close to 100% up to six months of use. After 12 months a small drop of about 10% for both knockdown and mortality rates was recorded. Despite this small drop and the constant loss of deltamethrin over time, these average rates were maintained up to three years with an average mortality and knockdown around or above 80%. Both LLINs performed significantly better than the CTNs after one year of use (GLMM mosquito mortality, P % 0.001) while after three years both LLIN average mortality rates did not differ (GLMM, P = 0.17). However when mortality rates over the entire three years are compared, PermaNet® 2.0 does obtain significantly higher mosquito mortality than Netprotect® (GLMM, P = 0.023). For each net type a multivariate analysis was carried out (GLMM) to assess the relative role of net use intensity, washing, net condition, dirtiness, chemical content and time of observation on the bio-efficacy of each net type. Time of observation showed a significant association with the bio-efficacy of all three net types (GLMM, P = 0.01, P % 0.001 and P = 0.006 respectively for CTN, Netprotect® and PermaNet® 2.0). CTN bio-efficacy was negatively correlated with bad net condition (GLMM, P = 0.03) and its chemical content (GLMM, P = 0.004), while for both LLINs net use intensity, number of washes, washing methods and the chemical content was not correlated to their corresponding bio-efficacy. For both Netprotect® and PermaNet® 2.0, the between- and within-net variations of knockdown and mortality follow similar trends (Table 3).

**Table 3:** **Cone bioassay results on *An. dirus *** **s.s.: Average mosquito knockdown (A) and mosquito mortality rates (B)**

**A**	**CTN**		**Netprotect®**		**PermaNet® 2.0**	
	**N**	**KD% (a)**	**N**	**KD% (a)**	**N**	**KD% (a)**
		**b**		**b**		**b**
**1 Week**	28	74.2 (28.7)	21	92.6 (14.5)	26	95.3 (10.4)
		13.2		5.3		4.3
**6 Months**	31	97.5 (8.0)	27	99.8 (1.9)	28	100
		2.1		0.3		
**12 Months**	28	56.3 (47.0)	26	84.4 (26.5)	27	88.4 (24.4)
		13.4		9.3		5.6
**18 Months**			29	91.8 (14.4)	27	93.5 (13.0)
				6.3		6.2
**24 Months**			29	78.8 (19.4)	30	85.7 (18.9)
				11.9		10.8
**30 Months**			25	85.0 (20.4)	28	86.5 (18.3)
				7.7		7.6
**36 Months**			24	81.7 (20.3)	27	87.2 (15.5)
				9.2		8.8
**B**	**CTN**		**Netprotect®**		**PermaNet® 2.0**	
	**N**	**Mortality% (a)**	**N**	**Mortality% (a)**	**N**	**Mortality% (a)**
		**b**		**b**		**b**
**1 Week**	28	81.5 (24.2)	21	98.2 (6.6)	26	99.2 (3.9)
		10.1		2.0		1.1
**6 Months**	31	97.3 (7.8)	27	99.5 (3.0)	28	100
		2.6		0.8		
**12 Months**	28	64.1 (48.4)	26	85.7 (23.3)	27	91.3 (18.1)
		12.5		9.5		6.0
**18 Months**			29	89.7 (17.6)	27	92.1 (17.1)
				8.2		7.6
**24 Months**			29	84.1 (22.9)	30	92.1 (13.4)
				9.4		6.8
**30 Months**			25	89.3 (16.3)	28	90.8 (14.9)
				6.5		6.2
**36 Months**			24	82.9 (25.0)	27	88.6 (15.2)
				9.6		8.9

CTN = conventionally-treated net, KD = knockdown, a = between net relative standard deviation (%), b = within-net variability (%).

The seven Netprotect® and four PermaNet® 2.0 nets that failed the bioassay criteria after 36 months were subjected to the tunnel test. All tunnel tests were valid, with an mortality in the control of less than 6% and more than 76% were blood fed. None of the tested nets passed the tunnel test criteria of 80% mortality or 90% blood feeding inhibition (see Additional file 5).

The proportions of LLINs that fulfil the WHO criteria (≥95% knockdown or ≥80% mortality) are reported in Table 4. Only 42% of the CTNs complied with these criteria after one year of use, while 73% and 85% of Netprotect® and PermaNet® 2.0 did. The proportion of Netprotect® nets that met the efficacy criteria varied throughout the study and was below the WHOPES threshold of 80% after 12, 24 and 36 months (respectively 73, 72 and 71%), however the 80% value was still within the confidence intervals. Whereas PermaNet® 2.0 performance was consistent over the entire three study years with a success rate of more than 80%. The acceptance of nets is entirely based on nets complying with the mortality criteria, as there are no nets that pass the 95% knockdown rate and then fail the 80% mortality rate. The acceptance over the period of one to three years was significantly higher for the reference LLIN than for Netprotect® (Mantel-Haenszel Test: OR = 2.091 [95% CI 1.071 – 4.083]; p = 0.0044).

**Table 4:** Percentage of nets meeting WHOPES criteria (knockdown ≥ 95% or mortality ≥ 80%) according to cone tests

	**CTN**		**Netprotect®**		**PermaNet® 2.0**	
	**N**	**%**	**N**	**%**	**N**	**%**
		**95% CI**		**95% CI**		**95% CI**
**1 Week**	28	60.7	21	100	26	100
		42.4 - 76.4		84.5 - 100		87.1 - 100
**6 Months**	31	100	27	100	28	100
		89 - 100		87.5 - 100		87.9 - 100
**12 Months**	28	42.9	26	73.1	27	85.2
		26.5 - 60.9		53.9 – 86.3		67.5 - 94.1
**18 Months**			29	89.7	27	92.6
				73.6 – 96.4		76.6 – 97.9
**24 Months**			29	72.4	30	93.3
				54.3 – 85.3		78.7 – 98.2
**30 Months**			25	88.0	28	85.7
				70 – 95.8		68.5 – 94.3
**36 Months**			24	70.8	27	85.2
				50.8 – 85.1		67.5 – 94.1

CTN = conventionally-treated net, CI = confidence interval.

After 36 months none the nets tested for the tunnel tests fulfil the WHOPES criteria.

## Discussion

This study evaluated the performance of Netprotect®, an interim recommended LLIN, using a WHOPES recommended LLIN, PermaNet® 2.0 as positive control, for three years under daily household use. A candidate net will be deemed to meet the requirements for an LLIN if, at the end of three years use, at least 80% of the sampled nets retain bio-efficacy (i.e. ≥95% mosquito knockdown rate or ≥80% mortality) with a standard WHO cone bioassay or with a tunnel test (≥80% mortality or ≥90% blood feeding inhibition) [7]. Following these criteria Netprotect® failed in the present phase III prospective study (71% of nets are effective after three years) and the LLIN status of PermaNet® 2.0 (85% of nets are effective after three years) was confirmed. Moreover Netprotect® failed for the bioassays criteria after already 12 and after 24 months (73% and 72% respectively of nets being effective).

However, the 95% confidence interval of the success rate for Netprotect® after three years is rather large (51-95%) and it still includes the 80% threshold. In the future, a more accurate estimate could be obtained by sampling more nets after three years of use. The new guidelines recommend sampling 50 nets after three years (instead of 30) [25] but this may still not be enough. However, throughout the three years the effectiveness of Netprotect® showed less consistency as compared to PermaNet® 2.0. Moreover over the entire observation period Netprotect® had a significant lower bio-efficacy as compared to PermaNet® 2.0. In terms of physical durability, Netprotect® performed as well as PermaNet® 2.0.

The marginal bio-efficacy performance of Netprotect® is probably related to the low insecticide content. At the baseline of the study already 43% of Netprotect® nets had a deltamethrin content below the target dose while PermaNet® 2.0 started with 27% of nets above their target dose showing that also recommended brands are outside of WHO specifications. The lower initial insecticide content for Netprotect® was probably a consequence of a problem in the manufacturing process as shown by a high proportion of the inactive *R*-alpha isomer (33%) relative to the deltamethrin content. Moreover, the proportion of the inactive *R*-alpha isomer, and so the isomerization, continued to increase throughout the study to up to 69% of deltamethrin content. As opposed to Netprotect®, PermaNet® 2.0 inactive deltamethrin *R*-alpha isomer remains almost constant during the 36 months of observation (3 to 7%).

In a similar phase III follow-up study of Netprotect® in Ghana [26], Netprotect® also failed the WHOPES requirements. Moreover, in the Ghana study, already after 18 months insufficient Netprotect® nets (63%) met the criteria. The bio-efficacy of Netprotect® showed also more inconsistency: after two years only 43% of Netprotect® nets passed the cone bioassay criteria while after 2.5 and 3 years respectively 64% and 62% (95% CI 48–74) did. Correspondingly in Ghana the average deltamethrin content of the Netprotect® nets at the baseline were at the lower limit of the specifications and with high amounts of the *R*-alpha isomer (30%). Cumulating the bioassay results of the sampled three-year old Netprotect® nets from both phase III studies in Ghana and Cambodia, only 64.9% (95% CI 54–75) of nets met the WHOPES criteria for a LLIN.

Based on different studies the 16th WHOPES working group meeting [26] recommended that until more evidence of Netprotect® is available from large scale studies, the WHO interim recommendation should be withdrawn and invite the national programs currently using Netprotect® to monitor efficacy and performance of this brand under local conditions [4]. Indeed, 80% of the nets did not fulfil the WHO criteria for bioassays after 3 years of use as prescribed in the Guidelines [7] applied for this evaluation. Moreover the specifications for the chemical content were not met.

The use of a fully recommended LLIN as positive control was shown to be extremely useful for validating the results of the study and is now being part of the new WHOPES guidelines [25]. However, before starting such three-year studies it would be essential that nets are checked for their compliance with WHO specifications and this for the candidate LLIN as well as for the reference net. This has now been adapted by the new WHOPES guidelines [25]. It would also be recommended to use confidence intervals for the proportion of nets meeting the WHO criteria for bioassays and to define a statistical approach for reviewing the bio-efficacy of LLINs over time.

## Competing interests

The authors declare that they have no competing interests.

## Authors’ contributions

MC and TS designed the study. KVR, VS and LD carried out the data analysis. KVR, LD and MC drafted the manuscript. OP carried out the chemical analysis using Gas Chromatography and contributed to interpretation of chemical data. MST did the bioassays. KVR, SS and TS facilitated and supervised the field work. VS, LD and MC critically reviewed the manuscript. All authors read and approved the final manuscript.

## Supplementary Material

Additional file 1 Household characteristics. Click here for file

Additional file 2 **Net use intensity. **The table shows the study participants declared net use intensity per survey and per study arm. Click here for file

Additional file 3 **Washing frequency.** The table shows the proportion of nets washed and the average frequency of washing per year. Click here for file

Additional file 4 **Physical condition of nets. **The table shows the average, median and interquartile range of the proportionate Hole Index of all three study arms and per survey. Proportion of nets in mediocre and poor condition is given. Click here for file

Additional file 5 **Tunnel test results on *An. dirus s.s*.: Average mosquito mortality rates and blood-feeding inhibition.** Click here for file
