# Supplementary material for: A phase III trial to evaluate the efficacy, fabric integrity and community acceptance of Netprotect® using a recommended long-lasting insecticidal net as positive control
Source: Malar J. 2014 Jul 7;13:256. doi: 10.1186/1475-2875-13-256 (PMC4105388; doi:10.1186/1475-2875-13-256)
Supplement: Additional file 1 — Household characteristics. [file 1475-2875-13-256-S1.docx]

**Additional file 1: Household characteristics**

|  | **N** | **%** |
| --- | --- | --- |
| **Total population size** | 3825 | 100 |
| **Gender** |  |  |
| M | 1861 | 48.7 |
| F | 1774 | 46.4 |
| Unknown | 190 | 5.0 |
| **Age distribution** |  |  |
| ≤ 5 | 525 | 13.7 |
| 6 - 16 | 1108 | 29.0 |
| 17 - 30 | 941 | 24.6 |
| 31 - 55 | 912 | 23.8 |
| ≥ 56 | 149 | 3.9 |
| Unknown | 190 | 5.0 |
| **Education Head of HH** |  |  |
| No | 527 | 69.2 |
| Primary | 166 | 21.8 |
| Secondary or more | 24 | 3.1 |
| Unknown | 45 | 5.9 |
| **Profession Head of HH** |  |  |
| None | 75 | 9.8 |
| Farmer | 625 | 82.0 |
| No farmer | 18 | 2.4 |
| Unknown | 44 | 5.8 |
| **House type** |  |  |
| Stilt | 628 | 82.4 |
| On the ground | 134 | 17.6 |
| **Wall structure** |  |  |
| Thatch | 163 | 21.4 |
| Wood | 596 | 78.2 |
| Brick | 3 | 0.4 |
| **Roof structure** |  |  |
| Thatch | 247 | 32.4 |
| Leave | 24 | 3.1 |
| Iron | 461 | 60.5 |
| Tile | 30 | 3.9 |
