# Supplementary material for: A phase III trial to evaluate the efficacy, fabric integrity and community acceptance of Netprotect® using a recommended long-lasting insecticidal net as positive control
Source: Malar J. 2014 Jul 7;13:256. doi: 10.1186/1475-2875-13-256 (PMC4105388; doi:10.1186/1475-2875-13-256)
Supplement: Additional file 2 — Net use intensity. The table shows the study participants declared net use intensity per survey and per study arm. [file 1475-2875-13-256-S2.docx]

**Additional file 2: Percentage of declared net use intensity per survey and per study arm (Number of HHs visited).**

| **Survey** | **Net use intensity** | **CTN** | **Netprotect**^®^ | **PermaNet^®^ 2.0** |
| --- | --- | --- | --- | --- |
| **1 (0.25 month)** | Year-round and every night | 93.3 (28) | 62.5 (20) | 83.9 (26) |
|  | Seasonally but every night |  | 6.3 (2) |  |
|  | Not used (new net) | 6.7 (2) | 28.1 (9) | 9.7 (3) |
|  | No answer |  |  | 3.2 (1) |
| **2 (3 months)** | Year-round and every night | 100 (32) | 84.4 (27) | 93.8 (30) |
|  | Seasonally but occasionally |  | 3.1 (1) |  |
|  | Not used (new net) |  | 3.1 (1) |  |
|  | No answer |  | 9.4 (3) | 6.3 (2) |
| **3 (6 months)** | Year-round and every night | 96.8 (30) | 87.1 (27) | 87.1 (27) |
|  | Year-round but occasionally | 3.2 (1) |  |  |
|  | Not used (new net) |  | 12.9 (4) | 9.7 (3) |
|  | No answer |  |  | 3.2 (1) |
| **4 (12 months)** | Year-round and every night | 82.2 (37) | 86.0 (117) | 89.4 (126) |
|  | Year-round but occasionally |  | 3.7 (5) |  |
|  | Seasonally but every night | 2.2 (1) | 0.7 (1) | 1.4 (2) |
|  | Seasonally but occasionally |  | 2.9 (4) | 0.7 (1) |
|  | Not used (new net) | 2.2 (1) | 1.5 (2) | 3.5 (5) |
|  | Presently not used | 4.4 (2) | 3.7 (5) | 3.5 (5) |
|  | No answer | 8.9 (4) | 1.5 (2) | 1.4 (2) |
| **5 (18 months)** | Year-round and every night |  | 73.3 (22) | 90.0 (27) |
|  | Year-round but occasionally |  | 10.0 (3) |  |
|  | Seasonally but occasionally |  | 6.7 (2) |  |
|  | Not used (new net) |  | 3.3 (1) | 6.7 (2) |
|  | Presently not used |  | 6.7 (2) | 3.3 (1) |
| **6 (24 months)** | Year-round and every night |  | 85.5 (53) | 100 (77) |
|  | Year-round but occasionally |  | 4.8 (3) |  |
|  | Seasonally but occasionally |  | 4.8 (3) |  |
|  | Not used (new net) |  | 3.2 (2) |  |
|  | No answer |  | 1.6 (1) |  |
| **7 (30 months)** | Year-round and every night |  | 17.2 (5) | 48.3 (14) |
|  | Not used (new net) |  | 3.4 (1) |  |
|  | Presently not used |  | 48.3 (14) | 20.7 (6) |
|  | No answer |  | 31.0 (9) | 31.0 (9) |
| **8 (36 months)** | Year-round and every night |  | 57.5 (15) | 60.0 (21) |
|  | Year-round but occasionally |  | 3.8 (1) |  |
|  | Seasonally but every night |  |  | 8.6 (3) |
|  | Presently not used |  | 34.6 (7) | 20.0 (7) |
|  | No answer |  | 3.8 (1) | 11.4 (4) |

CTN= conventionally treated net
