# Supplementary material for: A phase III trial to evaluate the efficacy, fabric integrity and community acceptance of Netprotect® using a recommended long-lasting insecticidal net as positive control
Source: Malar J. 2014 Jul 7;13:256. doi: 10.1186/1475-2875-13-256 (PMC4105388; doi:10.1186/1475-2875-13-256)
Supplement: Additional file 3 — Washing frequency. The table shows the proportion of nets washed and the average frequency of washing per year. [file 1475-2875-13-256-S3.docx]

**Additional file 3:**

**Washing frequency: Proportion of nets washed and the average frequency ‘washed nets’ are washed per year. After one week (Survey 1) none of the nets were washed.**

| **Survey** | **Net type** | **% of nets washed (Total Nbr)** | **Average number of washing times per year *** |
| --- | --- | --- | --- |
| **Year 1** | CTN | 44.4 (45) | 4.4 |
|  | Netprotect^®^ | 36 (136) | 3.4 |
|  | PermaNet^®^ 2.0 | 48.9 (141) | 3.5 |
| **Year 2** | Netprotect^®^ | 51.6 (62) | 1.7 |
|  | PermaNet^®^ 2.0 | 67.5 (77) | 2.1 |
| **Year 3** | Netprotect^®^ | 26.9 (26) | 1.3 |
|  | PermaNet^®^ 2.0 | 57.1 (35) | 2.4 |

*This is the average wash frequency per year for nets that are washed at least once.

CTN= conventionally treated net
