# Supplementary material for: A phase III trial to evaluate the efficacy, fabric integrity and community acceptance of Netprotect® using a recommended long-lasting insecticidal net as positive control
Source: Malar J. 2014 Jul 7;13:256. doi: 10.1186/1475-2875-13-256 (PMC4105388; doi:10.1186/1475-2875-13-256)
Supplement: Additional file 4 — Physical condition of nets. The table shows the average, median and interquartile range of the proportionate Hole Index of all three study arms and per survey. Proportion of nets in mediocre and poor condition is given. [file 1475-2875-13-256-S4.docx]

**Additional file 4: Physical condition of nets.**

| **Net Type** | **Months** | **N** | **Average**  **pHI** | **Median**  **pHI hole index** | **Interquartile range pHI (0.25 - 0.75)** | **Proportion of nets in mediocre and poor condition in %  (pHI > 175)** |
| --- | --- | --- | --- | --- | --- | --- |
| **CTN** | 0.25 | 28 | 8.0 | 0 | 0 - 0 | 0 |
|  | 6 | 31 | 43.4 | 2 | 0 – 69.0 | 6.5 |
|  | 12 | 28 | 115.7 | 59 | 8.3 - 136.5 | 21.4 |
| **Netprotect^®^** | 0.25 | 21 | 0.9 | 0 | 0 - 0 | 0 |
|  | 6 | 27 | 4.0 | 0 | 0 - 2.5 | 0 |
|  | 12 | 30 | 7.4 | 0.5 | 0 - 2.8 | 0 |
|  | 18 | 29 | 41.8 | 12 | 0 – 70.0 | 0 |
|  | 24 | 29 | 48.6 | 26 | 6.0 – 60.0 | 6.9 |
|  | 30 | 25 | 95.8 | 33 | 1.0 – 140.0 | 16.0 |
|  | 36 | 24 | 175.2 | 67.5 | 14.0 - 185.8 | 25.0 |
| **PermaNet^®^ 2.0** | 0.25 | 26 | 0.7 | 0 | 0 - 0 | 0 |
|  | 6 | 28 | 20.4 | 0 | 0 - 0.3 | 3.6 |
|  | 12 | 27 | 58.5 | 3 | 0 – 27.0 | 7.4 |
|  | 18 | 27 | 26.1 | 3 | 0 - 32.5 | 3.7 |
|  | 24 | 30 | 63.5 | 21.5 | 7.0 – 84.0 | 16.7 |
|  | 30 | 28 | 134.9 | 50 | 11.0 – 101.0 | 21.4 |
|  | 36 | 27 | 164.3 | 127 | 40.5 - 219.5 | 29.6 |

pHI: proportionate hole index, CTN= conventionally treated net
