# Supplementary material for: A phase III trial to evaluate the efficacy, fabric integrity and community acceptance of Netprotect® using a recommended long-lasting insecticidal net as positive control
Source: Malar J. 2014 Jul 7;13:256. doi: 10.1186/1475-2875-13-256 (PMC4105388; doi:10.1186/1475-2875-13-256)
Supplement: Additional file 5 — Tunnel test results on An. dirus s.s.: Average mosquito mortality rates and blood-feeding inhibition. [file 1475-2875-13-256-S5.docx]

**Additional file 5: Tunnel test results on *An. dirus s.s*.: Average mosquito mortality rates and blood feeding inhibition for each individual net that failed the cone bioassays after 3 years.**

| **Net type** | **Mortality Control (%)** | **Blood Feeding control (%)** | **Mortality Tunnel test (%)** | **Blood Feeding Inhibition (%)** |
| --- | --- | --- | --- | --- |
| **Netprotect^®^** | 5 | 77 | 16.84 | 41.56 |
|  | 3 | 90 | 61.86 | 72.22 |
|  | 1 | 81 | 24.49 | 45.68 |
|  | 3 | 85 | 70.10 | 88.24 |
|  | 0 | 92 | 23.0 | 50.0 |
|  | 1 | 76 | 62.63 | 61.84 |
|  | 3 | 91 | 64.95 | 78.02 |
| **PermaNet^®^ 2.0** | 1 | 92 | 32.32 | 67.39 |
|  | 1 | 91 | 37.37 | 71.43 |
|  | 1 | 92 | 32.32 | 70.65 |
|  | 0 | 85 | 62.5 | 26.47 |
